# Evaluating remote post-mortem veterinary meat inspections on pig carcasses using pre-recorded video material

**DOI:** 10.1186/s13028-023-00678-x

**Published:** 2023-04-05

**Authors:** Viktor Almqvist, Charlotte Berg, Arja Helena Kautto, Jan Hultgren

**Affiliations:** 1 Department of Animal Environment and Health, Swedish University of Agricultural Sciences, P.O. Box 234, Skara, 532 23 Sweden; 2 Department of Biomedical Sciences and Veterinary Public Health, Swedish University of Agricultural Sciences, P.O. Box 7036, Uppsala, 750 07 Sweden

**Keywords:** Abattoir, Agreement, Digital video communication, Official Veterinarian, Slaughter, Remote inspection

## Abstract

**Background:**

Official meat inspections at small-scale slaughterhouses and game-handling establishments in geographically remote areas place a heavy burden on the meat-producing food business operators. By performing meat inspections remotely using live-streamed video, instead of on-site, the official control could meet the goals of sustainability, resilience and logistics. We investigated the agreement between the two approaches at pig slaughter. Two official veterinarians (OVs) inspected 400 pig carcasses at a Swedish slaughterhouse, with each pig being inspected on site by one OV and remotely by the other. After a period of 3 to 6 months, video recordings of the remote inspections were assessed again by the same OVs, thus enabling direct comparisons of previous on-site inspections and renewed video-based inspections within the same OV.

**Results:**

Agreement across 22 finding codes was generally very high for both OVs. In all but one case (whether to fully condemn a carcass), for both OVs, Prevalence-Adjusted Bias-Adjusted kappa was well above 0.8, indicating ‘almost perfect agreement’.

**Conclusions:**

This study supports earlier findings that reliable post-mortem inspections can be performed using video, and indicates higher agreement between remote and on-site inspections if the same OV performs both.

## Background

All animals processed at slaughterhouses and game-handling establishments designated to the common market in the European Union (EU) must undergo regulated on-site controls according to EU regulations [1]. These inspections are carried out under the supervision or responsibility of an official veterinarian (OV). Flexibility in the regulations creates the opportunity for official auxiliaries (OA) and specifically designated control personnel to perform certain tasks. Rules in force demanding ante- and post-mortem inspections (AMI, PMI) to be performed on site for each animal, even in low-capacity establishments in remote areas, create large problems from a sustainability, resilience and logistical point of view. For example, the slaughter of 50,000 reindeer in 12 different abattoirs during 2021 gave rise to 60,000 km of car travel for the control personnel [2]. This is not in line with the United Nations’ Agenda 2030 [3] or the European Green Deal [4], nor with Swedish official sustainability goals [5].

Both AMI and PMI focus on food safety, animal health and animal welfare. Only healthy animals are accepted for slaughter. PMI takes place after the carcass has been degutted and split. The term ‘carcass’ is henceforth used to denote the entire carcass together with accompanying organs.

In Sweden, a specific code system is used for the documentation of findings at PMI, made up of two-digit codes representing the most commonly occurring and important symptoms or findings [6]. Carcasses are inspected by an OA, and any carcasses with suspicious findings are separated from the main line and additional examination is carried out by an OV before a final decision is reached.

With the expansion of 4G and 5G mobile infrastructure, and even fibre-based internet connection, together with advances in video encoding and transmission, PMI at remote sites via video link could become a sustainable alternative to current routines of on-site personnel. This would reduce travel by allowing control personnel to perform inspections at small-scale establishments from a centrally located office. Live video applications have been researched and implemented in human medicine and are currently used in e.g. internet-based medical consultations [7] and various surgical procedures [8, 9]. The technique is also used in clinical veterinary medicine [10], with several developers active in the market.

We have previously shown promising results when evaluating remote PMI, comparing it to on-site PMI, but observed some differences between the two methods [11]. As two different OVs were used in the evaluations (one using each method), any inter-method differences might have been masked by systematic differences in assessments between the OVs. Ideally, the same veterinarian would perform both the on-site and remote inspection of the same carcasses, with sufficient separation in time so as not to remember the initial inspection. However, this is hardly possible when assessing carcasses during on-going slaughter. In addition, most studies on meat inspection would have to be carried out in a real-life setting at a commercial slaughter plant, placing further constraints on what could be achieved in practice. If the same OV could perform PMI both on site and via video transmission with sufficient time in between, this potential problem of difference between OVs could be circumvented. In our previous study we recorded all remote PMI [11], and through the use of these video files, an OV could perform PMI via video on the same carcasses as those previously inspected on site, after a couple of months’ time to reduce recognition memory. By comparing the results of these video inspections to prior results from on-site PMI, the problem of using multiple OVs could be largely mitigated.

The aim of this study was to assess the inter-method, intra-rater reliability between remote and on-site PMI when both are performed by the same OV, by comparing results of inspections performed on video-records of remote, live-video PMI to previous records from PMI performed on site, separately for two OVs.

## Methods

### Data collection

This study is based in part on previously produced material [11]. Two veterinarians (OVA and OVB), each with several years’ experience of working as OVs, inspected 400 carcasses arrested by OAs for further inspection, using on-site inspection and performing the inspections remotely with the aid of a technician on site. The technician presented the carcass through video, performed any manual tasks the OV deemed necessary, and relayed any requested information back to the OV. Randomly selected falsely detained carcasses (no findings) formed a negative control group of 220 carcasses. The OVs switched methods during the study, inspecting 200 carcasses with each method.

The remote inspections were conducted using video-call software supplemented with augmented reality (Remote Guidance, XMReality AB, Linköping, Sweden), which visualised the carcasses. Each remote PMI was recorded, including the video feed, augmented-reality overlays (when applicable), and all audio communication between the OV and the on-site technician. The videos were stored as h.264 encoded video files with a resolution of 720 × 1280p30, at 3 Mbit/s, a bitrate hard-capped by the XMReality software site [11]. Each video file contained the PMI of a single carcass, totalling 400 videos.

**Fig. 1:**
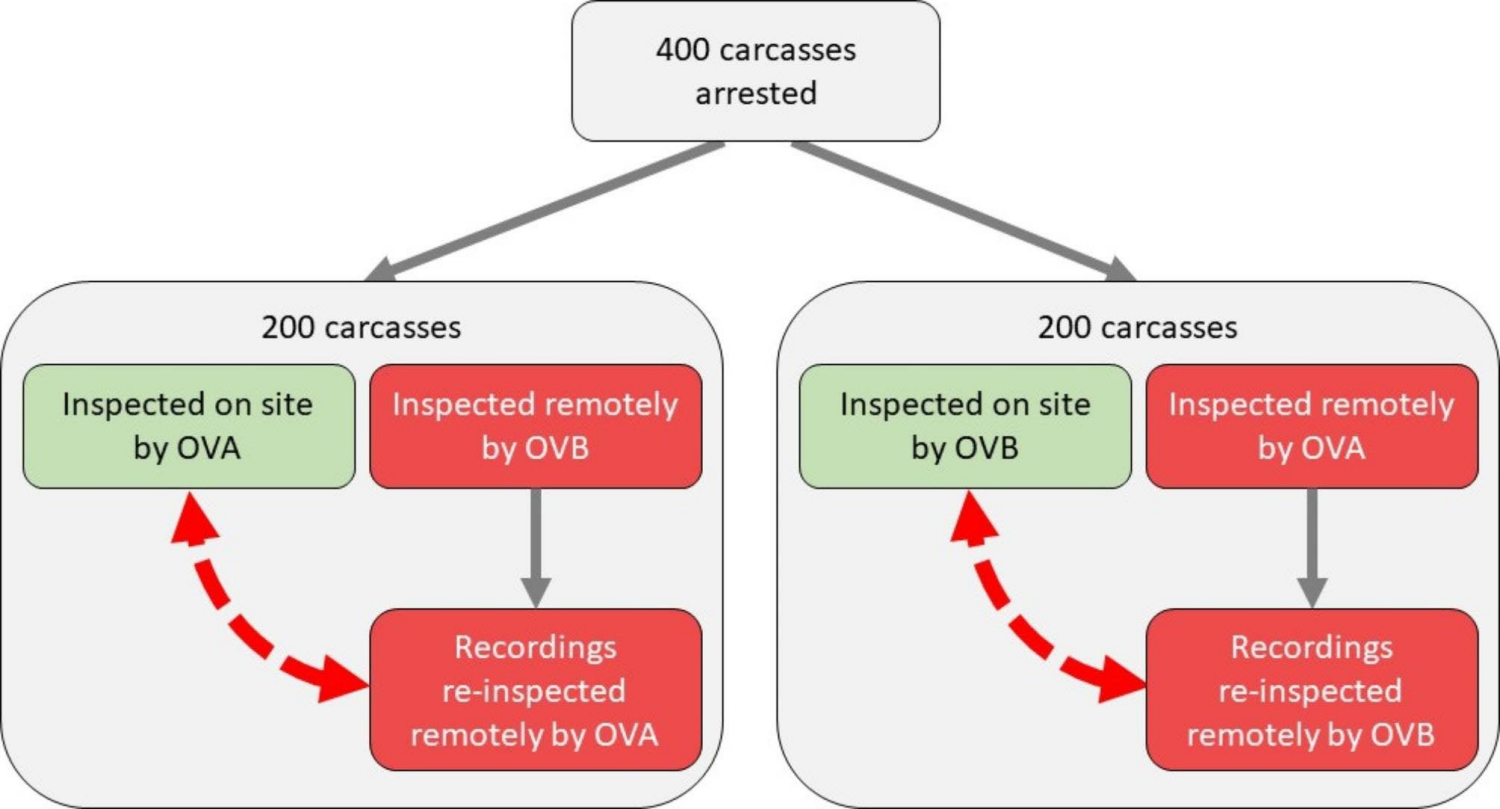
Schematic representation of comparisons of on-site to recorded video material of post-mortem meat inspection performed by two official veterinarians (OVs).

The video recordings were reviewed by OVA and OVB, with each of them reviewing 200 videos produced by the other, i.e., OVA assessed the videos produced by OVB, and vice versa. A schematic overview of the inspections is detailed in Fig. 1.

Three to six months had passed between the on-site inspections and the review of the videos, to reduce recognition memory. Average video length was 6 min 35 s for the OVB videos viewed by OVA, and 4 min 45 s for the OVA videos viewed by OVB. As a side-note, average time for on-site PMI of the same carcasses was 1 min 53 s [11]. PMI findings were recorded using a modified version of the instruction issued by the Swedish Food Agency [6], to which code 56 for ‘kidney lesion’ (which is normally not recorded in pigs) and code 999 to denote ‘no findings’ were added (Table 1).

**Table 1:** Codes for documentation of findings in post-mortem inspections *of pig carcasses*

Code	Finding	Code	Finding	Code	Finding
06	Atypical mycobacteriosis	40	Old injury	76	Pleuritis and/or endocarditis
18	Erysipelas	42	Recent injury	78	Pleuritis and peritonitis
19	Systemic infectious disease	48	Emaciation	84	Parasitic liver lesions, “white spots”
26	Tumour	52	Other finding^b^	88	Other liver lesions
30	Abscess	56	Kidney lesion	999	No findings
32	Arthritis	58	Tail lesion	FA	Perceived falsely arrested
34	Abnormal appearance	62	Swine enzootic pneumonia	TC	Totally condemned
36	PSE^a^	64	Other pneumonia		
38	Fatty liver	72	Actinobacillus pleuropneumonia		

^a^ Quality condition characterised by pale, soft and exudative meat

^b^ Code used to denote conditions with no individual code, in this study predominantly splenic torsion

The list of findings is modified from instructions for meat inspection in Sweden [6] by the addition of codes FA, ‘perceived false arrest’; TC, ‘totally condemned’; 56, ‘kidney lesion’; and code 999 to denote ‘no findings’

For each inspection, all codes were stored as binary variables (present or not). The codes represented common lesions or conditions, along with two classifications made by the OV, i.e. perceived false arrest (FA) and total condemnation (TC), where FA indicated that the inspector considered the carcass to have been falsely arrested by the OA and TC that the OV considered the carcass to be unfit for human consumption. The FA and TC classifications were based on the findings at PMI and were mutually exclusive, i.e., a carcass perceived as FA could not be TC, and vice versa. The OA were instructed that only carcasses without any findings be arrested falsely. The OVs also recorded their perceived certainty about the inspection result on a Likert scale from 1 (not at all confident) to 5 (completely confident). The findings and certainty obtained for the video inspections were then compared with data for on-site inspections of the same 200 carcasses by the two OVs from our previous study [11].

### Statistical analysis

For both OVs and for each finding or classification, prevalence, observed percentage agreement (joint probability of agreement), Cohen’s kappa [12], Prevalence-Adjusted Bias-Adjusted Kappa (PABAK) [13] and indices of prevalence and bias were calculated, as per recommendations [14] along with 95% confidence intervals. Since no single measure was believed to be an exhaustive representation of agreement, the values were used together to assess the degree of agreement between on-site and video inspection for both OVs. Kappa-based agreement is measured on a scale of 0–1, with a suggested 5-step interpretation, with 0.01–0.20 representing “None to slight agreement”, 0.21–0.40 “Fair agreement”, 0.41–0.60 “Moderate agreement”, 0.61–0.80 “Substantial agreement” and 0.81–1.00 “Almost perfect agreement” [15].

Prevalence was calculated as the average number of reports of a certain finding in both on-site and video inspections, divided by the number of inspected carcasses (n = 200). Calculations on TC were based on the subset of carcasses assessed as not FA during both inspections (n = 79 for OVA, n = 83 for OVB).

All statistical calculations and analyses were performed in R [16]. Cohen’s kappa and PABAK were calculated using the function epi.kappa() in the package epiR [17].

## Results

The two OVs scored PABAK values above 0.8 in 21 (OVA) and 19 (OVB) out of the 22 evaluated findings. When comparing PABAK scores between the OVs finding for finding, OVA produced better overall agreement than OVB. For codes 58 (tail lesion) and 84 (parasitic liver lesions), however, OVB scored 0.08 and 0.05 higher, respectively, than OVA. Both OVs produced PABAK values lower than 0.8 for code 999 (no finding; 0.77 and 0.75 for OVA and OVB, respectively). In addition, OVB scored 0.75 for code 56 (kidney lesion) and 0.44 for TC (total condemnation). For OVA, there was a slight increase in average certainty, from 4.32 to 4.51, when comparing on-site to video inspection, while OVB instead showed a marked decrease, from 4.61 to 3.17. For both OVs there was a substantial difference between registrations of FA (falsely arrested) and code 999, with roughly 75% of FA carcasses also bearing code 999.

Detailed results for OVA and OVB are presented in Tables 2 and 3.

**Table 2:** Intra-rater reliability measurements per finding for OVA.

Finding (Code or classification)	Prevalence, %	Cohen’s kappa	PABAK	Observed agreement, %
Atypical mycobacteriosis (06)	1.00	1.00 (0.86–1.00)	1.00 (0.96–1.00)	100
Systemic infectious disease (19)	4.50	0.77 (0.63–0.90)	0.96 (0.9–0.99)	98.0
Tumour (26)	0.00	0.00 (0.00–0.00)	1.00 (0.96–1.00)	100
Abscess (30)	4.00	0.87 (0.73–1.01)	0.98 (0.93–1.00)	99.0
Arthritis (32)	5.00	0.89 (0.76–1.03)	0.98 (0.93–1.00)	99.0
Abnormal appearance (34)	0.50	0.00 (0.00–0.00)	0.98 (0.93–1.00)	99.0
PSE (36)	1.75	0.57 (0.44–0.69)	0.97 (0.91–0.99)	98.5
Old injury (40)	1.00	1.00 (0.86–1.00)	1.00 (0.96–1.00)	100
Recent injury (42)	0.50	1.00 (0.86–1.00)	1.00 (0.96–1.00)	100
Emaciation (48)	0.50	1.00 (0.86–1.00)	1.00 (0.96–1.00)	100
Other finding (52)	2.00	0.75 (0.61–0.88)	0.98 (0.93–1.00)	99.0
Kidney lesion (56)	9.50	0.65 (0.51–0.79)	0.88 (0.8–0.94)	94.0
Tail lesion (58)	22.5	0.80 (0.66–0.94)	0.86 (0.77–0.92)	93.0
Swine enzootic pneumonia (62)	9.50	0.65 (0.52–0.79)	0.88 (0.8–0.94)	94.0
Other pneumonia (64)	23.0	0.94 (0.81–1.00)	0.96 (0.9–0.99)	98.0
Actinobacillus pleuropneumonia (72)	0.50	1.00 (0.86–1.00)	1.00 (0.96–1.00)	100
Pleuritis and/or endocarditis (76)	14.0	0.88 (0.74–1.01)	0.94 (0.87–0.98)	97.0
Parasitic liver lesions, “white spots” (84)	7.25	0.44 (0.31–0.58)	0.85 (0.76–0.91)	92.5
Other liver lesions (88)	0.25	0.00 (0.00–0.00)	0.99 (0.94–1.00)	99.5
No findings (999)	46.3	0.77 (0.63–0.91)	0.77 (0.66–0.85)	88.5
Perceived falsely arrested (FA)	59.8	0.97 (0.83–1.11)	0.97 (0.91–0.99)	98.5
Totally condemned (TC)	18.4	0.71 (0.49–0.92)	0.82 (0.65–0.93)	91.1

Estimated prevalence, inter-method reliability based on Cohen’s kappa [12] (with 95% confidence interval), prevalence- and bias-adjusted kappa (PABAK [13]; with 95% confidence interval) and observed percentage agreement for individual finding codes (n = 200), FA (‘perceived false arrest’, n = 200) and TC (‘total condemnation’, n = 79) in comparisons between recorded remote and on-site post-mortem inspections of pig carcasses at a Swedish slaughterhouse in 2019 by an official veterinarian *(OVA).*

**Table 3:** Intra-rater reliability measurements per finding for OVB.

Finding (Code or classification)	Prevalence, %	Cohen’s kappa	PABAK	Observed agreement, %
Atypical mycobacteriosis (06)	1.50	0.66 (0.52–0.80)	0.98 (0.93–1.00)	99.0
Systemic infectious disease (19)	4.50	0.54 (0.41–0.67)	0.92 (0.85–0.97)	96.0
Tumour (26)	0.50	1.00 (0.86–1.00)	1.00 (0.96–1.00)	100
Abscess (30)	5.75	0.86 (0.72–1.00)	0.97 (0.91–0.99)	98.5
Arthritis (32)	4.00	0.74 (0.60–0.88)	0.96 (0.90–0.99)	98.0
Abnormal appearance (34)	5.25	0.35 (0.23–0.48)	0.87 (0.78–0.93)	93.5
PSE (36)	2.00	1.00 (0.86–1.00)	1.00 (0.96–1.00)	100
Old injury (40)	0.25	0.00 (0.00–0.00)	0.99 (0.94–1.00)	99.5
Recent injury (42)	1.00	0.50 (0.38–0.62)	0.98 (0.93–1.00)	99.0
Emaciation (48)	1.00	0.00 (0.00–0.00)	0.96 (0.90–0.99)	98.0
Other finding (52)	8.00	0.93 (0.79–1.07)	0.98 (0.93–1.00)	99.0
Kidney lesion (56)	16.8	0.56 (0.43–0.69)	0.75 (0.64–0.83)	87.5
Tail lesion (58)	20.5	0.91 (0.77–1.05)	0.94 (0.87–0.98)	97.0
Swine enzootic pneumonia (62)	10.0	0.61 (0.48–0.75)	0.86 (0.77–0.92)	93.0
Other pneumonia (64)	25.0	0.81 (0.67–0.95)	0.86 (0.77–0.92)	93.0
Actinobacillus pleuropneumonia (72)	1.50	0.66 (0.53–0.79)	0.98 (0.93–1.00)	99.0
Pleuritis and/or endocarditis (76)	15.0	0.76 (0.63–0.90)	0.88 (0.80–0.94)	94.0
Parasitic liver lesions, “white spots” (84)	12.8	0.75 (0.61–0.89)	0.89 (0.81–0.94)	94.5
Other liver lesions (88)	1.75	0.27 (0.14–0.41)	0.95 (0.89–0.98)	97.5
No findings (999)	37.8	0.74 (0.60–0.87)	0.75 (0.64–0.83)	87.5
Perceived falsely arrested (FA)	55.0	0.86 (0.72–1.00)	0.86 (0.77–0.92)	93.0
Totally condemned (TC)	29.5	0.37 (0.18–0.56)	0.45 (0.23–0.63)	72.3

Estimated prevalence, inter-method reliability based on Cohen’s kappa [12] (with 95% confidence interval), prevalence- and bias-adjusted kappa (PABAK [13]; with 95% confidence interval) and observed percentage agreement for individual finding codes (n = 200), FA (‘perceived false arrest’, n = 200) and TC (‘total condemnation’, n = 83) in comparisons between recorded remote and on-site post-mortem inspections of pig carcasses at a Swedish slaughterhouse in 2019 by an official veterinarian (OVB).

## Discussion

When comparing on-site to remote PMI within OVs, both of the two showed higher levels of agreement for almost all findings and classifications than previously observed in comparison of inspection methods across OVs [11]. In all but one code for OVA (code 36, PSE) and five findings for OVB (codes 34, abnormal appearance; 48, emaciation; 88, other liver lesion; FA and TC), PABAK values were higher than in our previous study. Five of the six PABAK values for OVB were only marginally lower than previously [11] and the values were well above 0.8 in both studies. Small differences such as these have very little impact when using kappa-based statistics, due to the rather large steps on the interpretation scale used for kappa [15]. The only substantial difference between this study and previous results was the PABAK value for TC found for OVB, which was 0.44 in this study, compared to 0.50 [11]. The same was true for Cohen’s kappa and percentage agreement, with a large majority of the findings showing higher agreement in this study than previously obtained [11]. We have previously argued that the results of a switch from on-site to remote inspections would have less effect than switching between two OVs on-site [11], and this is underpinned by the fact that agreement in general was even higher in the present study.

Some resulting values for Cohen’s kappa were either 1 or 0. These were considered artefacts, most likely deriving from the extremely low prevalence of the relevant findings (in most cases less than 1%). Due to the overall good health of pigs at slaughter in Sweden it is difficult to produce a sample with high prevalences of all findings. In this study the sample consisted of all carcasses arrested for in-depth inspection, and the prevalences of some findings were still very low. In order to acquire a meaningful absolute number of carcasses with these rare findings the size of the experiment would increase many times over. A sample consisting of pre-selected carcasses would have to be constructed, which would be unfeasible purely because of decomposition of the material over time; by the time you had a large enough sample of rare findings, the first would have degraded. If some findings are rare, mathematically the impact on consumers of poor agreement between methods would be low, since there are so few carcasses that would be affected in absolute numbers. Additionally, it has been suggested that very few findings at PMI are considered hazardous to consumers [18].

In our previous study we made an initial assumption that the two OVs were equally skilled at the start of the study, which was in part contradicted by the results [11]. The present study supports that OVA and OVB cannot be considered completely equal in terms of PMI performance, and substantiates the claim [11] that previous differences between on-site and remote PMI are, at least partly, attributable to the individual OV. Although data collection was standardised, the OVs may still have differed slightly in their inspection routines in time spent, details focused on and manner of decision making. Thus, performing PMI according to someone else’s routine might open up for poor performance. For example, if OVB was very thorough and took the time to inspect in detail, while OVA was quicker to draw a conclusion, when the inspections were viewed by the other OV, OVA would benefit from the extra thoroughness, whereas OVB would be restricted by the shorter videos produced by OVA. This could explain why, when the finding TC scored 0.50 comparing on-site to remote PMI [11], in this study one OV scored 0.45 and the other 0.82, a very marked difference. Had OVB inspected videos of their own on-site inspections the scored would likely have been much higher. The impact of remote PMI, and any shortcomings in agreement between it and on-site PMI has been thoroughly discussed previously [11]. To further expand on this, the results for OVA in the present study shows that remote PMI can actually display “almost perfect agreement” in terms of PABAK across all but one finding (with the last one being close) evaluated both here and previously. This fact alone additionally strengthens the hypothesis that on-site and remote PMI can be interchangeable, assuming a thorough and systematic inspection routine.

It has been noted that longer, more thorough video inspections lead to higher accuracy in human video diagnostics [19]. The pre-recorded PMI videos produced by OVB were on average almost 2 min longer than those produced by OVA, and OVA was more certain when reviewing the videos than during on-site inspections, while OVB was substantially less certain of the video assessments of OVA’s videos. In fact, if the videos that OVB reviewed were too short or otherwise not sufficiently thorough compared with on-site inspection of the same carcasses, this could have contributed to the relatively poor agreement of TC classifications for OVB. If remote PMI was performed in real time, where the OVs could directly affect the inspection routine, the results would likely improve, at least for OVB, who was probably at a disadvantage when reviewing the shorter inspection videos produced by OVA, as discussed above. The differences in inter-method reliability observed between the two OVs highlight the importance of a standardised, thorough inspection routine for remote inspections.

Most findings had similar (but not identical) estimated prevalences for the two OVs. Kappa-based statistics are sensitive to the prevalence of findings, which could have contributed to the observed differences in agreement between the OVs. It cannot be assumed that the distribution of findings was the same for the OVs, since they inspected different carcasses, but it should be similar enough for these differences to be small.

In this study, PABAK showed very good agreement between the methods for both OVs, with only four values below 0.8. However, the relevance of agreement with low prevalence of findings can be questioned, even when using PABAK, since most agreement would stem from negative cases. In our previous results we found that the agreement between OVs tends to be lower for findings that are more subjective, rather than objective assessments [11]. This subjectivity could be said to apply to all findings with PABAK below 0.8 in this study as well.

Another explanation for the lack of perfect agreement between the methods could be that there is some variation, or a random element, in how a person classifies the same finding at repeated inspections, i.e. test-retest agreement. It is not unreasonable to expect a certain degree of variation since no inspector can be assumed to perform completely consistently. The importance of noise; variation in daily individual variation in decision making has been pointed out [20], and differences in experience and knowledge, and in opinion, motivation and dedication, may explain differing agreement between meat inspectors [21]. Motivation and dedication can most likely also vary within an individual, which could cause variations in agreement in this type of comparison. A suitable follow-up study would be test-retest evaluation of the material and the OVs, in order to determine the magnitude of this variation. The discrepancies between registrations of FA and code 999 (which would ideally have been the same) could perhaps also be attributed to these explanations [20, 21]; it is possible the OVs were overly motivated and thorough, being part of a research project, and the OAs were simply performing their normal day-to-day tasks as usual.

This study was primarily based on kappa statistics [12]. Even if the suggested classification of kappa from 0.01 to 0.20 (“none to slight agreement”) to 0.81-1.00 (“almost perfect agreement”) [15] has been criticised as slightly rough and arbitrary [22] it is still the accepted standard. Cohen’s kappa is primarily sensitive to varying prevalence, with low prevalence tending to lower the kappa values, although percentage agreement remains unchanged [13, 23–26]. It has been pointed out that kappa always assumes a fixed prior probability of rating, either positive or negative [23], and that it always assumes total randomness in the chance agreement [27]. It is likely that most people who guess would at least attempt to make an educated guess rather than “flip a coin”, and the assumption of total randomness is therefore not correct. Instead it can be reasoned that the “true agreement” is probably somewhere between Cohen’s kappa and the observed percentage agreement [27]. We previously concluded that PABAK statistic seems to fit neatly with this criterion [11], and it was therefore used in the present study as well. However, due to the core differences between different agreement measures, we considered it important to report all three values (Cohen’s kappa, PABAK and percentage agreement), in order to give a nuanced picture of the agreement.

## Conclusions

This study supports previous findings that the kappa-based agreement between post-mortem inspections of pig carcasses on-site during slaughter and remotely afterwards using video recordings is “almost perfect”. The study also indicates that remote inspections show better agreement when comparisons are made with the same official veterinarian performing both inspections.

## Data Availability

The data that support the findings of this study are available from the Swedish Food Agency but restrictions apply to the availability of these data, which were used under license for the current study, and hence they are not publicly available. Data are however available from the authors upon reasonable request and with permission of the Swedish Food Agency.

## Declarations

### Prior publication

The data included in this article have, in part, previously been published in a Doctoral thesis *Who you gonna call? Examining the possibilities of remote veterinary meat inspection*, by *Almqvist, V, Uppsala 2021.*

## References

[1] Regulation, EU) 2017/625 of the European Parliament and of the Council of 15 March 2017 on official controls and other official activities performed to ensure the application of food and feed law, rules on animal health and welfare, plant health and plant protection products, amending Regulations (EC) No 999/2001, (EC) No 396/2005, (EC) No 1069/2009, (EC) No 1107/2009, (EU) No 1151/2012, (EU) No 652/2014, (EU) 2016/429 and (EU) 2016/2031 of the European Parliament and of the Council, Council Regulations (EC) No 1/2005 and (EC) No 1099/2009 and, Directives C. 98/58/EC, 1999/74/EC, 2007/43/EC, 2008/119/EC and 2008/120/EC, and repealing Regulations (EC) No 854/2004 and (EC) No 882/2004 of the Eu-ropean Parliament and of the Council, Council Directives 89/608/EEC, 89/662/EEC, 90/425/EEC, 91/496/EEC, 96/23/EC, 96/93/EC and 97/78/EC and Council Decision 92/438/EEC (Official Controls Regulation)Text with EEA relevance. OJ. 2017;L95:1–142.

[2] Kautto AH. Remote Meat Control – from opportunity to obligation? In: RIBMINS Scientific Meeting 7, 2022. https://ribmins.com/wp-content/uploads/2022/04/7_4_2022_4_Arja-Helena-Kautto.pdf. Accessed 9 April 2022.

[3] United Nations. The Sustainable Development Agenda. https://www.un.org/sustainabledevelopment/development-agenda. Accessed July 5 2022.

[4] European Commission. European Green Deal. https://ec.europa.eu/clima/eu-action/european-green-deal_en. Accessed 5 July 2022.

[5] Government Offices of Sweden. The Global Goals and the 2030 Agenda for Sustainable Development. https://www.government.se/government-policy/the-global-goals-and-the-2030-Agenda-for-sustainable-development. Accessed 5 July 2022.

[6] Swedish Food Agency. Kontroller vid slakt [Controls at slaughter]. https://www.livsmedelsverket.se/produktion-handel--kontroll/livsmedelskontroll/offentlig-kontroll/kontroller-vid-slakt. Accessed 5 March 2022.

[7] Schroeder C (2019). Pilot study of telemedicine for the initial evaluation of general surgery patients in the clinic and hospitalized settings. Surg Open Sci.

[8] Marescaux J, Leroy J, Rubino F, Smith M, Vix M, Simone M (2002). Transcontinental Robot-Assisted remote telesurgery: feasibility and potential applications. Ann Surg.

[9] Wang SC, Singh TP (2017). Robotic repair of a large abdominal intercostal hernia: a case report and review of literature. J Robotic Surg.

[10] Oxley J, Saunders R (2015). Potential for telemedicine. Companion Anim.

[11] Almqvist V, Berg C, Hultgren J (2021). Reliability of remote post-mortem veterinary meat inspections in pigs using augmented-reality live-stream video software. Food Control.

[12] Cohen J (1960). A coefficient of Agreement for Nominal Scales. Educ Psychol Meas.

[13] Byrt T, Bishop J, Carlin JB (1993). Bias, prevalence and kappa. J Clin Epidemiol.

[14] Sim J, Wright CC (2005). The Kappa Statistic in Reliability Studies: use, Interpretation, and sample size requirements. Phys Ther.

[15] Landis JR, Koch GG (1977). The measurement of Observer Agreement for categorical data. Biometrics.

[16] R Core Team. R: a language and environment for statistical computing (2021.09.0). R foundation for statistical computing, Vienna, Austria. 2022. https://www.R-project.org/.

[17] Stevenson M. Evan Sergeant with contributions from Telmo Nunes, Cord Heuer, Jonathon Marshall, Javier Sanchez, epiR: Tools for the Analysis of Epidemiological Data. R package version 2.0.19. https://CRAN.R-project.org/package=epiR

[18] Hill A, Brouwer A, Donaldson N, Lambton S, Buncic S, Griffiths I. A risk and benefit assessment for visual-only meat inspection of indoor and outdoor pigs in the United Kingdom. Food Control., Löw S, Erne H, Schütz A, Eingartner C, Spies CK. The required minimum length of video sequences for obtaining a reliable interobserver diagnosis in wrist arthroscopies. Arch Orthop Trauma Surg. 2015;135:1771–7. 10.1007/s00402-015-2339-y 26423659

[19] Kahneman D, Sibony O, Sunstein CR (2021). Noise: a flaw in human judgment.

[20] Stärk KDC, Alonso S, Dadios N, Dupuy C, Ellerbroek L, Georgiev M (2014). Strengths and weaknesses of meat inspection as a contribution to animal health and welfare surveillance. Food Control.

[21] McHugh ML (2012). Interrater reliability: the kappa statistic. Biochem Med (Zagreb).

[22] Feinstein AR, Cicchetti DV (1990). High agreement but low Kappa: I. the problems of two paradoxes. J Clin Epidemiol.

[23] Di Eugenio B, Glass M (2004). The Kappa Statistic: a second look. Comput Linguist Assoc Comput Linguist.

[24] Nelson KP, Edwards D (2008). On population-based measures of agreement for binary classifications. Can J Statistics.

[25] Hallgren KA (2012). Computing Inter-Rater Reliability for Observational Data: an overview and Tutorial. Tutor Quant Methods Psychol.

[26] Zhao X, Liu JS, Deng K (2013). Assumptions behind Intercoder Reliability Indices. Ann Int Commun Assoc.

